# Investigating the Role of Thrombosis, Fenestration, and False Lumen Orbital Orientation in the Hemodynamics of Type B Aortic Dissection

**DOI:** 10.21203/rs.3.rs-3997160/v1

**Published:** 2024-03-15

**Authors:** Joseph C. E. Messou, Kelly Yeung, Eric Sudbrook, Jackie Zhang, Shahab Toursavadkohi, Areck A. Ucuzian, Eleonora Tubaldi

**Affiliations:** 1 Department of Electrical and Computer Engineering, University of Maryland, College Park, MD 20742, USA; 2 Fischell Department of Bioengineering, University of Maryland, College Park, MD 20742, USA; 3 Department of Mechanical Engineering, University of Maryland, College Park, MD 20742, USA; 4 Division of Vascular Surgery, Department of Surgery, University of Maryland, Baltimore, MD 21201, USA; 5 Center for Vascular & Inflammatory Diseases, University of Maryland, Baltimore, MD, 21201, USA; 6 Baltimore VA Medical Center, Vascular Service, Baltimore, MD, 21201, USA; 7 Division of Cardiology, College of Medicine, University of Maryland, Baltimore, MD 21201, USA; 8 Robert E. Fischell Institute of Biomedical Devices, University of Maryland, College Park, MD 20742, USA

## Abstract

While much about the fundamental mechanisms behind the initiation and progression of Type B aortic dissection (TBAD) is still unknown, predictive models based on patient-specific computational fluid dynamics (CFD) can help in risk stratification and optimal clinical decision-making. Aiming at the development of personalized treatment, CFD simulations can be leveraged to investigate the interplay between complex aortic flow patterns and anatomical features. In this study, the hemodynamics of false lumen thrombosis, a large fenestration, and the orbital orientation of the false lumen is studied through image-based CFD simulations on three TBAD patient-specific geometries. A new pipeline was developed leveraging the open-source software SimVascular and Paraview to analyze multiple patients simultaneously and to achieve large-scale parallelization in CFD results based on patients’ computed tomography (CT) images. The results of this study suggest that the internal orbital orientation of the false lumen contributes to maintaining a positive luminal pressure difference $\Delta P_{TL-FL}=P_{TL}-P_{FL}$ between the true lumen (TL) and the false lumen (FL), despite an impingement area in the false lumen near the entry tear. A positive and high luminal pressure difference is thought to promote TL expansion and FL compression. Moreover, it was also found that both FL thrombosis at the entry tear region, and the presence of a large fenestration in the descending thoracic aorta reduce the magnitude of the negative luminal pressure difference, which in turn may reduce FL expansion and the risk of unstable aortic growth.

## Introduction

Acute Type B aortic dissection (TBAD) is a life-threatening condition caused by a primary tear in the aortic intima, which allows blood to flow through the aortic media. This results in aortic wall delamination and in the creation of a false lumen (FL) of blood flow. This secondary flow channel propagates longitudinally beside the original channel (i.e., true lumen, TL)^1,2^, with the two lumens separated by a dissection flap. If left untreated, TBAD can lead to fatal complications such as chronic aneurysmal degeneration or acute organ malperfusion due to branch vessel ischemia^3^. With modern medical and surgical therapy, an increasing number of patients survive the TBAD acute state and enter the chronic phase of the disease where continued lifelong monitoring is needed to minimize the risk of morbidity and mortality. Surveillance imaging plays a pivotal role in assessing chronic TBAD disease progression and detecting late complications, such as aneurysm formation^4,5^. Currently, the maximal aortic diameter is the principal determinant of long-term risk of rupture, though it is likely to be insufficient in precisely predicting future adverse events^6,7^. Hemodynamic features, such as wall shear stress (WSS), oscillatory shear index (OSI), peak velocity, flow rate, and FL retrograde flow, could help identify early indicators of FL expansion and potential rupture.

Computational fluid dynamics (CFD) is a powerful tool to determine complex intra-aortic hemodynamics typical of TBAD patients^8,9^. Image-based CFD simulations can predict the distribution of near-wall hemodynamic parameters with high-resolution in arteries^10^. CFD patient-specific modeling and parametric studies have been successfully performed to explore the effects of specific parameters, such as boundary conditions^11^, which affect the complex blood flow regime and flow-induced wall stresses^12–14^. Recent CFD studies reported that FL growth correlates with low time-averaged WSS values in the distal false lumen^15,16^, while in the region of the tear, high blood velocity and WSS values correlate with disease progression via FL dilation^16–18^. Moreover, it was observed that a decreased velocity gradient at the entry tear, which results in a decreased time-averaged WSS, prevents the vessel from rupture^19^. Fewer fenestrations have been shown to potentially represent a predictor for unstable aortic growth^20^. Similarly, a negative luminal pressure difference $\left(\Delta P_{TL-FL}=P_{TL}-P_{FL}\right)$ is often associated with the expansion of the false lumen and the compression of the true lumen, while a positive and high pressure difference is thought to protect the true lumen from compressing^21,22^. Recently, four-dimensional flow magnetic resonance imaging (4D Flow-MRI) has been increasingly used to provide non-invasive direct measurements of TBAD blood flow regimes^23,24^. Despite the limited spatial and temporal resolutions, 4D Flow-MRI has a growing role in informing CFD boundary conditions^25,26^ and in validating CFD results^27^.

CFD simulations can also be leveraged to assess the effectiveness of treatment approaches and plan surgical interventions for TBAD^28^. For instance, evaluating the impact of stent grafts after thoracic endovascular aortic repair (TEVAR) has the potential to become a practical pre-surgical tool^22,29^. In addition, aortic fenestration is an uncommon but possibly effective procedure for treating acute aortic dissection, by redirecting blood flow into the true lumen^30^. However, there is currently not enough data to justify the routine use of the fenestration technique. Thus, CFD simulations can emerge as an important tool to properly select patients that would benefit from this treatment, as an alternative to aortic replacement^31,32^. Finally, CFD simulations may provide insights into the hemodynamic consequences of fenestration coverage by endografts during TEVAR to the FL, TL, and distal organ vessels such as the mesenteric arteries. Overall, the state-of-the-art for CFD simulations of aortic dissection is rapidly evolving, helping clinicians better understand the complex hemodynamics and, simultaneously, optimizing treatment outcomes.

Inspired by the growing role of CFD prediction models in advancing TBAD risk stratification and personalized care^8,33^, this study analyzes the morphological and hemodynamic indices of three patients with different entry tear locations and false lumen orbital orientations. Patient-specific fluid models are created to simulate physiological blood flow conditions. In addition, in order to investigate how a large fenestration in the descending thoracic aorta and FL thrombosis affect TBAD hemodynamics, we compare three versions of the same patient’s dissection considering (i) a large fenestration and a false lumen with thrombosis (original model), (ii) a primary entry tear and a false lumen without thrombosis nor fenestration, and (iii) a primary entry tear, a large fenestration, and a false lumen without thrombosis.

## Methods

### Patient-specific geometries

**Figure 1.**
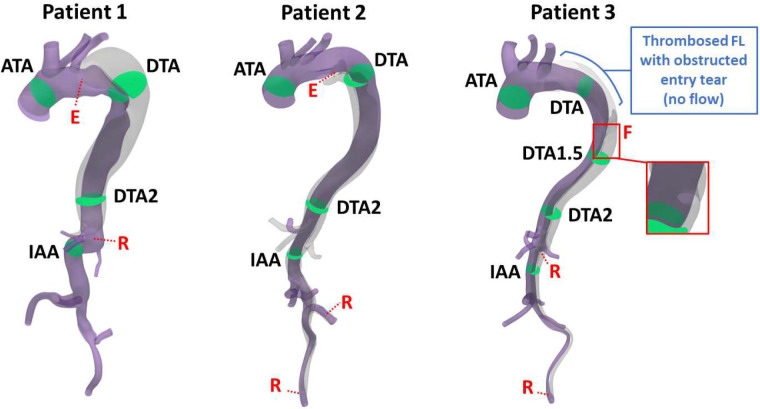
Patient-specific fluid models with red labels denoting the entry tear (E), re-entry tear (R), and fenestration (F) locations. The following cross-sections at areas of interest are shown in green: ascending thoracic aorta (ATA), descending thoracic aorta (DTA/DTA1.5/DTA2), intra-abdominal aorta (IAA). Patient 3’s large fenestration of 3.7 mm (red box) is located between the DTA and DTA2 cross-sections. The true and false lumens are shown in purple and grey, respectively. The location of the thrombus (i.e. no flow) in the false lumen (FL) of Patient 3 is highlighted in blue.

This study was reviewed and approved by the University of Maryland, Baltimore, Institutional Review Board (IRB). The need for informed consent was waived by the IRB of the University of Maryland, Baltimore. All data acquisition and methods were performed in accordance with relevant guidelines and regulations. Computer tomography (CT) angiography images of the chest, abdomen, and pelvis of three patients suffering from Type B aortic dissection were obtained per standard of care at the University of Maryland Medical Center using a Siemens SOMATOM Force CT scanner (see details in Table 1) and retrospectively analyzed. The high-resolution CT images (ranging from 0.74 × 0.74 mm/pixel to 0.98 × 0.98 mm/pixel) were used to reconstruct the anatomic model of the dissected aorta for each patient (Fig. 1).

While all patients present a Type $B_{3,x}$ aortic dissection^34^, the geometry and size of the entry tear, as well as the distal extent of the dissection differ as reported in Table 1. We refer to fenestrations at the distal extent of the dissection or in a branch as re-entry tears. Patient 1 presents a large entry tear (12.1 mm) while Patient 2’s entry tear is relatively small (5.4 mm). As shown in Figure 1, Patient 3’s entry tear is obstructed by a thrombus in the false lumen that ends in the descending thoracic aorta. Patient 1’s dissection extends to the paravisceral abdominal aorta, and the only re-entry tear is located distal to the celiac trunk. Patient 2’s dissection extends into the abdomen, where the re-entry tears are located at the level of the left internal and external iliac arteries. While in Patient 1 the FL runs on the outer curvature of the aortic arch, in Patient 2 it runs on the inner curvature. Patient 3’s dissection extends into the abdomen, where the re-entry tears are located in the aorta at the level of the left renal artery and the left external iliac artery. Several of the abdominal branch arteries are also dissected and the FL runs on the outer curvature of the aortic arch. The patient also has a large fenestration (3.7 mm) in the descending thoracic aorta, between the two cross-sectional areas DTA and DTA2 as shown in Figure 1.

### CFD Model and Mesh Generation

**Figure 2.**
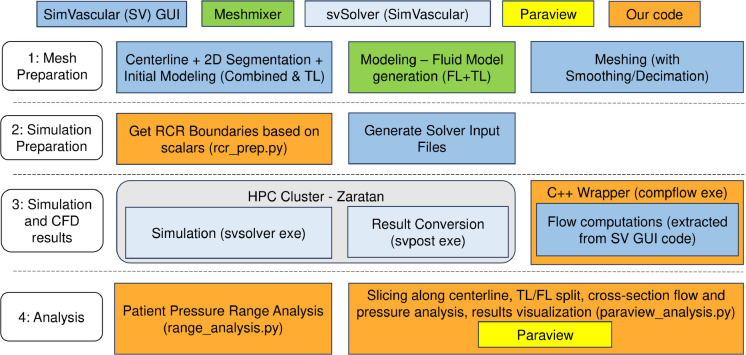
Overall pipeline from a patient’s CT images to the final CFD results. The code written to accelerate the pipeline and simplify the analysis of multiple patients is highlighted in orange.

As shown in Figure 2, SimVascular^35^, an open-source cardiovascular modeling and simulation software, was used to segment the CT images, model the fluid domain, and simulate blood flow through the patient-specific geometries. Adopting the methodology outlined by Bäumler *et al.*^36^, the CT images were segmented to construct two separate surface models using SimVascular: one model with the TL alone and another including the TL, FL, and dissection flap, called the combined model. These models were exported to Meshmixer (Autodesk, Inc.), where the fluid domain was generated. First, the TL model was extruded assuming a uniform wall thickness of 2 mm. Boolean operations were used to generate the FL model by subtracting the extruded model from the combined model. The FL and original non-extruded TL were combined at the entry and re-entry tears to form the fluid domain, with a uniform dissection flap separating the TL and FL. The model was smoothed and re-meshed in Meshmixer before being exported to SimVascular for further smoothing operations with the goal of smoothing the bifurcations.

Due to the location of the entry tear in Patient 2 and the fenestration in Patient 3 (Fig. 1), this method was only applied from the bottom of the entry tear and fenestration to the iliac arteries. The upper part of the fluid domain was obtained separately in SimVascular by (1) segmenting the true lumen up to the tear, (2) segmenting the false lumen up to the tear, (3) segmenting the merged true and false lumens at the tear, and (4) combining 1, 2, and 3. The fluid model from the inlet to the tear and the fluid model from the bottom of the tear to the iliac arteries were then merged in Meshmixer before being imported back into SimVascular for further smoothing operations. For each patient, tetrahedral meshes of the fluid domain with a maximum edge size of 2 mm, 1.5 mm, 1 mm, and 0.8 mm were created with the TetGen mesh generator^37^ embedded in SimVascular to numerically solve partial differential equations using a finite element-based solver^38^ and test for mesh convergence. Mesh convergence was observed for a maximum edge size of 1 mm for all patients, and the final meshes had 6.1M, 4.8M, and 4.9M tetrahedral elements for Patient 1, 2, and 3, respectively. Final simulations were run for 1000 time steps per cardiac cycle with a step size of 0.779 ms to match a cardiac cycle period of 0.779 seconds. Cycle-to-cycle periodicity was reached within 4–5 cycles, and results are reported on the last cycle. As a reference, 1000 steps for a mesh of 4.8M tetrahedral elements ran approximately for 6h on a compute node with 128 cores (dual AMD 7763 64-core CPUs). A fluid density $\rho_{f}=0.00106\;g/{mm}^{3}$ and a fluid viscosity $\mu_{f}=0.004g/(mm\cdot s)$ were used.

### Inlet Flow Rates and Outlet RCR Boundary Conditions

**Figure 3.**
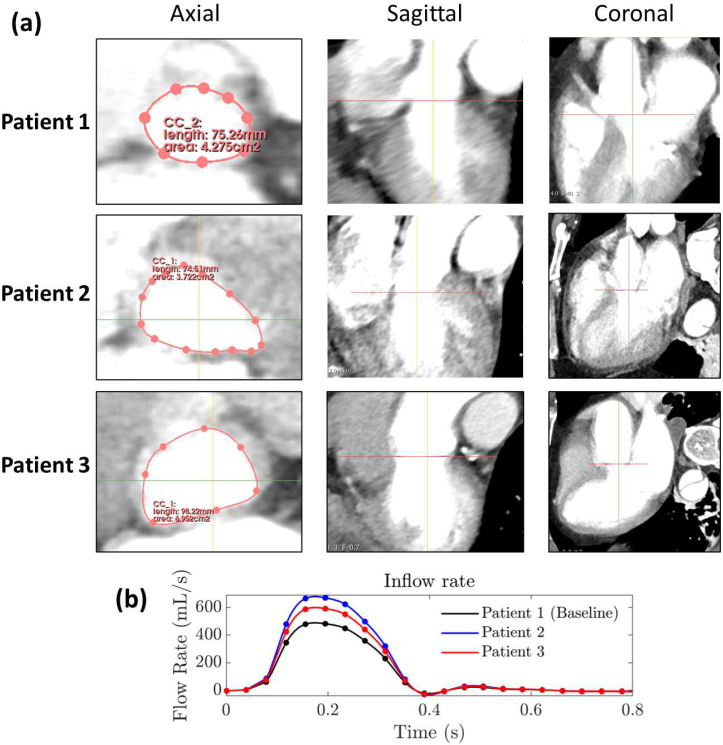
**(a)** CT images showing the LVOT Areas of Patient 1 (4.275 *cm*^2^), 2 (3.722 *cm*^2^), and 3 (6.962 *cm*^2^). The images are rotated so that the axial view is perpendicular to blood flow in the sagittal view. **(b)** Imposed inlet flow rate over one cardiac cycle (*t* = 779 ms). The cardiac outputs of Patient 2 and 3 are used to scale the baseline inlet flow from Bäumler *et al.*^36^, which corresponds to a stroke volume of 96.5 mL.

A parabolic inlet flow waveform^36^ was tuned to match the patient-specific stroke volume when available (Fig. 3). For Patient 2 and 3, patient-specific stroke volume was derived using the CT images and echocardiogram reports. Using the Left Ventricular Outflow Tract (LVOT) Velocity Time Integral (VTI) from the echocardiogram reports and the LVOT area computed by direct planimetry from the CT images (Fig. 3), the stroke volume (SV) was computed as $SV(L)=LVOT\_VTI\;(cm)*LVOT\_Area\;({cm}^{2})$^39^. Using this method yields stroke volumes of 134.0 mL (LVOT VTI = 36 cm, LVOT Area = 3.722 c*m*^2^) and 118.4 mL (LVOT VTI = 17 cm, LVOT Area = 6.962 c*m*^2^) for Patient 2 and 3, respectively. As the LVOT VTI of Patient 1 was not recorded, the patient-specific stroke volume could not be computed. Therefore, we used the default stroke volume of 96.5 mL from Bäumler *et al.*^36^, where the false lumen shape is similar to Patient 1. As outlet conditions for each branch *j*, a three-element Windkessel model (or RCR) was applied using $q_{j}$, the fractional flow rate at the outlet obtained from Baulmer *et al.*^36^ (Supplementary, Table S1). The flow-proportional outlet resistance and capacitance are estimated by $R_{T,j}=R_{T}/q_{j}$ and $C_{j}=C_{T}\cdot q_{j}$, respectively, where $R_{T}$ is the total resistance and $C_{T}$ is the total capacitance. For each outlet, the distal and proximal resistance can be written as $R_{d,j}=k_{d}\cdot R_{T,j}$ and $R_{p,j}=\left(1-k_{d}\right)R_{T,j}$, respectively, with $k_{d}=0.9$ being the ratio of distal to total resistance^36,40^. The Windkessel parameters were tuned for each patient on a medium-size mesh in order to obtain a systolic blood pressure $\left(P_{sys}\right)$, a diastolic blood pressure $\left(P_{dia}\right)$, a pulse pressure $\left(P_{sys}-P_{dia}\right)$, and a mean arterial pressure $\left(\left(P_{sys}+2*P_{dia}\right)/3\right)$ within 10.8% of the patient-specific pressures (Supplementary, Table S2).

### Efficient Pipeline for Tuning Parameters

While SimVascular has an advanced graphical user interface (GUI) to compute the pressure and flow rate at the inlet and outlets, using the GUI can become a bottleneck when running large-scale experiments. In particular, depending on the target pressure tolerance, parameter tuning can take up to 10 runs of the numeral solver for a single patient. This involves (1) using the GUI to compute the pressure and flow rate at the faces (inlet and outlets), (2) comparing the obtained pressure to the patient-specific blood pressure, (3) generating a new set of outlet boundary conditions, and (4) running the solver with the new set of parameters. As shown in Figure 2, we developed scripts to efficiently tackle the aforementioned steps 1 to 3. *Complow* is a C++ wrapper that uses the open-source code from the SimVascular GUI to compute the pressure and flow rate at the faces without any user intervention. This allows the simultaneous analysis of any number of files as long as enough computing resources can be allocated. Once the executable has completed the run, the pressure range analysis script (range_analysis.py) can automatically run in sequence and compare the results to the patient-specific blood pressure. The user can then look at the relative differences and tune the parameters accordingly. Once the new scaling factors of the total resistance and capacitance are obtained, the user can then use the rcr_prep.py script to generate the new RCR boundary conditions. As the number of patients studied grows, using the combination of these executable and scripts will considerably improve the user workflow.

### CFD Solver Results Analysis

**Figure 4.**
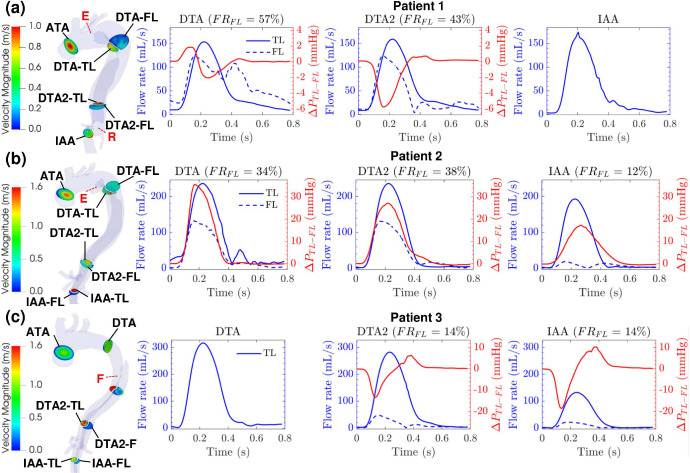
Patient-specific models with cross-sections of interest colored by velocity magnitude at peak systole (t = 234 ms) for **(a)** Patient 1, **(b)** Patient 2, and **(c)** Patient 3. Red labels denote the location of the entry tear (E), re-entry tear (R), and large fenestration (F). The graphs show patients’ TL flow rate (solid blue line), FL flow rate (dashed blue line), luminal pressure difference $\Delta P_{TL-FL}$ (red line), and FL flow ratio $FR_{FL}$ at cross-sections of interest (DTA, DTA2, and IAA) over one cardiac cycle.

Once the final set of parameters has been chosen and the final CFD solver results are obtained, the next step is to visualize and analyze the data. To perform this efficiently while being consistent across multiple patients, we developed a script based on Paraview^41,42^, as shown in Figure 2. The script can be configured to automatically generate figures for the fluid models, such as WSS and OSI distributions, and velocity streamlines. In addition, given the centerline of the fluid model extracted from SimVascular, the script automatically makes slices perpendicular to the centerline and saves the coordinates of the slices. The user can then select the cross-sections of interest or process all slices. A feature specific to aortic dissection models was also added: the automatic detection of slices with two regions. When two regions are found in a slice, the script analyzes each region separately, stores the results as a large region and a small one, and writes a file listing all small regions. The user can then update that file and confirm if each small region corresponds to the false lumen or not before converting the results from large and small regions to TL and FL regions. The regions can then be visualized as shown in the models of Figure 4 or processed to compute the flow rate and the mean pressure that are shown in the plots of Figure 4. Overall, this script accelerates the visualization and processing of the CFD results, which is highly beneficial when multiple patients are involved in the study.

## Results

### Flow Rate and Pressure Distributions

Figures 4(a–c) show results at different cross-sections over a cardiac cycle for Patient 1, 2, and 3, respectively. The graphs show the TL and FL blood flow rates $Q_{TL}$ and $Q_{FL}(mL/s)$, the luminal pressure difference $\Delta P_{TL-FL}(mmHg)=P_{TL}-P_{FL}$, and the FL flow ratio $FR_{FL}(\%)=100*\left({\overset{‾}{Q}}_{FL}/\left({\overset{‾}{Q}}_{TL}+{\overset{‾}{Q}}_{FL}\right)\right)$, with $\overset{‾}{Q}$ denoting the average flow rate over time. The cross sections of interests are: the ascending thoracic aorta (ATA), descending thoracic aorta (DTA/DTA2), and intra-abdominal aorta (IAA).

In Patient 1 (Fig. 4.a), the luminal pressure difference $\Delta P_{TL-FL}$ is minimal at DTA, but decreases before systole at DTA2, where the pressure in the FL is higher than the one in the TL by up to 5.8 mmHg. The FL flow rate is either higher than the TL flow rate or at least 80% of it at all times, with a FL flow ratio $FR_{FL}$ of 57% at DTA and 43% at DTA2. This is consistent with the large entry tear (12.1 mm) and the size of the dilated FL. The FL area is 3.4x larger at DTA and 2.9x larger at DTA2 compared to the TL area.

In Patient 2 (Fig. 4.b), all cross sections present very high luminal pressure difference $\Delta P_{TL-FL}:35.6\;mmHg,27.1\;mmHg,\;17.3\;mmHg$ are found around peak systole at DTA, DTA2, and IAA, respectively. The TL flow rate is higher compared to the FL flow rate, and the cross-sectional areas are relatively similar except at DTA where the FL area is 2.8x smaller than the TL area. Notably, the flow rate in the FL at IAA is much lower when compared to all other cross-sections. The FL flow ratio $FR_{FL}$ is 34%, 38%, and 12% at DTA, DTA2 and IAA, respectively.

In Patient 3 (Fig. 4.c), the luminal pressure difference $\Delta P_{TL-FL}$ is low at DTA2 (−13.4 mmHg) and IAA (−18.6 mmHg) before peak systole, then increases during diastole up to 6.3 mmHg and 10.3 mmHg at DTA2 and IAA, respectively. While the FL and TL areas are comparable at DTA2, the FL flow rate is much lower than the TL flow rate, with a FL flow ratio $FR_{FL}$ of 14%. The FL flow ratio stays the same at IAA despite the FL area becoming 0.6x smaller than the TL area.

For all the patients, the velocity magnitude at all cross-sections is higher in the true lumen than in the false lumen except at the DTA cross-section of Patient 2 where both lumens have comparable velocity magnitudes (Fig. 4).

### Velocity Streamlines

**Figure 5.**
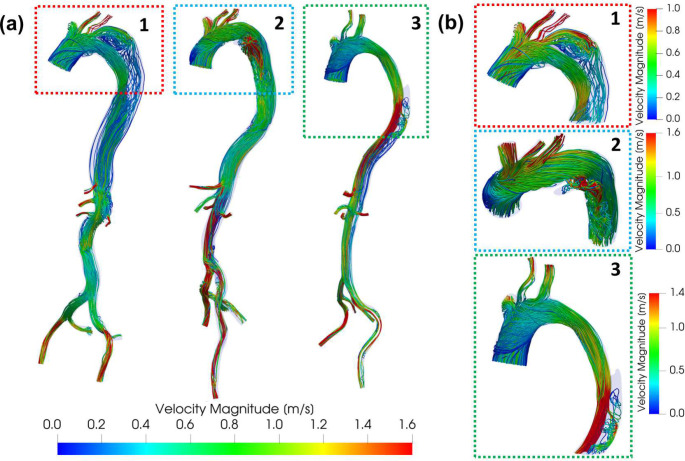
**(a)** Patients’ velocity streamlines colored by velocity magnitude at peak systole (t = 234 ms). **(b)** Zoomed-in views of the aortic arch and entry tear/large fenestration showing: (i) a flow jet through the entry tear/large fenestration and (ii) a recirculating flow in the FL. A helical flow can also be observed in the FL of Patient 2 and 3.

The velocity streamlines at peak systole (*t* = 234 ms) of patient models with a zoom on the flow behavior at the entry tear and large fenestration regions are shown in Figure 5. A high-velocity flow jet that creates local recirculating flow in the FL is observed through the entry tear of Patient 1 and 2 and the large fenestration of Patient 3. Additionally, in both Patient 2 and 3, helical flow patterns are present in the FL distal to the entry tear and fenestration. In Patient 1, the FL flow velocity peak is found in the impingement area of the flow jet on the FL outer curvature wall. Similarly, the flow jet in Patient 2 creates an impingement area on the FL inner curvature wall. No impingement area is found in Patient 3. While the velocity of the flow in the FL increases in the vicinity of the tear and fenestration for all patients, it reduces as the flow progresses down the thoracic aorta where it is much lower than the velocity of the flow in the true lumen.

### TAWSS and OSI

**Figure 6.**
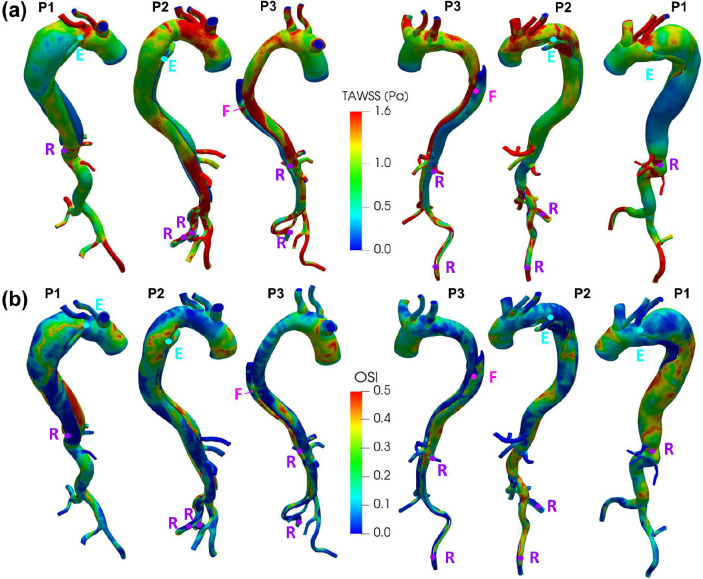
**(a)**TAWSS distribution and **(b)** OSI distribution for Patient 1 (P1), Patient 2 (P2), and Patient 3 (P3). (E) and (R) mark the entry and re-entry tear(s) locations, (F) marks the location of a large fenestration in the descending thoracic aorta of Patient 3.

Time averaged wall shear stress (TAWSS), a measure of shear stress acting on the endothelial cells lining of the aortic wall averaged over the heartbeat cycle, and oscillatory shear index (OSI), a dimensionless parameter quantifying the level of deviation of the WSS from its average direction, were computed for all patients as shown in Figure 6.a and Figure 6.b, respectively. In Patient 1, locally-high TAWSS values are observed in the proximity of the entry tear and re-entry tear regions. The FL impingement area of the flow jet through the entry tear corresponds to locally-high TAWSS. In the thoracic descending portion, the FL presents a general trend of low TAWSS and high OSI, and the opposite is found for the TL. In Patient 2, the FL presents a higher TAWSS in the thoracic aorta and it decreases in the abdominal aorta before increasing again in the iliac arteries. The FL impingement area of the flow jet through the entry tear corresponds to high TAWSS. In the TL, low TAWSS values are observed until the abdominal portion is reached. A peak in the OSI values is found in the TL in the vicinity of the entry tear and in the FL in left external iliac artery. In Patient 3, the FL presents low TAWSS and high OSI distributions throughout the thoracic descending dissected portion. In the TL, TAWSS is high at the curvature of the descending aorta next to the large fenestration while OSI is high at the end of the curvature. The large fenestration presents a moderately high TAWSS and OSI.

### Hemodynamic effects of Patient 3’s Entry Tear Models

**Figure 7.**
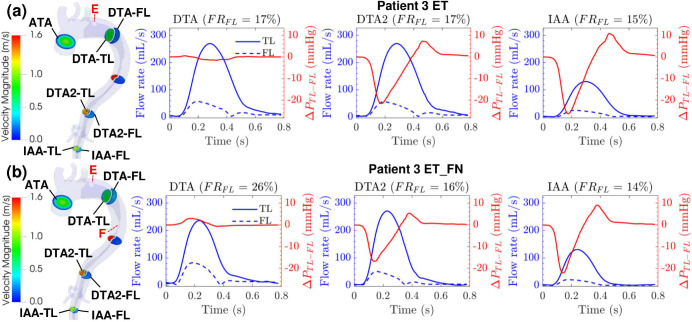
**(a)** Patient 3’s ET (entry tear only) and **(b)** ET_FN (entry tear and fenestration) models with cross-sections of interest colored by velocity magnitude at peak systole (t = 234 ms), and red labels denoting the location of the entry tear (E) and large fenestration (F). The graphs show the TL flow rate (solid blue line), FL flow rate (dashed blue line), luminal pressure difference $\Delta P_{TL-FL}$ (red line), and FL flow ratio $FR_{FL}$ at cross-sections of interest (DTA, DTA2, and IAA) over one cardiac cycle.

**Figure 8.**
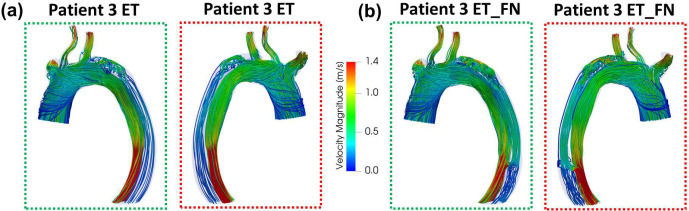
**(a)** ET model and **(b)** ET_FN model of Patient 3 velocity streamlines colored by velocity magnitude at peak systole (t = 234 ms).

To further investigate how a large fenestration in the descending thoracic aorta and the presence of a thrombus in the FL impact the TBAD hemodynamics, two additional anatomic models were created for Patient 3. These models reflect the wide variability in the pathologic anatomic features seen in acute aortic dissection and syndromes. In both models, the thrombosed FL is considered as a part of the fluid domain with a primary entry tear of size 11 mm × 9 mm. As shown in Figure 7, the original fenestration is removed in the first model (ET), and both the primary entry tear and the same large fenestration as the original model are considered in the second model (ET_FN). As shown in Figure 7, the new anatomic models of Patient 3 (ET and ET_FN) both present a minimal luminal pressure difference $\Delta P_{TL-FL}$ at DTA, while the FL flow rate is higher in the ET_FN model. At DTA2 and IAA, the luminal pressure difference $\Delta P_{TL-FL}$ and flow rate of the new models (ET and ET_FN) follow similar trends as the original model of Patient 3 (Fig. 4). However, the range of the luminal pressure difference $\Delta P_{TL-FL}$ decreases from the ET model to the ET_FN model, to the original model. Before peak systole, the FL pressure is higher than the TL pressure at DTA2 by 21.6 mmHg, 16.7 mmHg, and 13.4 mmHg in the ET, ET_FN, and original models, respectively . The same trend is observed at IAA with a FL pressure higher than TL pressure by 26.3 mmHg, 22.1 mmHg, and 18.6 mmHg for the ET, ET_FN, and original models, respectively. While the FL flow ratio $FR_{FL}$ at the DTA cross-section is 9% lower in the ET model with respect to the ET_FN model, it becomes comparable at the DTA2 and IAA cross-sections. The velocity streamlines for the ET and ET_FN models are shown in Figure 8. A higher-velocity flow jet is present through the entry tear of the ET_FN model which creates a stronger recirculating flow and an impingement area in the FL distal to the entry tear. At the fenestration in the ET_FN model, the flow goes from the FL to the TL differently with respect to the original model where we observe a flux from the TL to the FL (Figures 5 and 8). For the TAWSS and OSI distributions, all Patient 3 models follow a similar trend distal to the fenestration. Both ET and ET_FN models present an impingement area with high TAWSS in the proximity of the entry tear; higher TAWSS values are observed in the ET_FN model (Supplementary, Figure S1).

## Discussion

In the present study, hemodynamic parameters for three TBAD patients have been determined through patient-specific CFD simulations. All patients have an entry tear located distal to the left subclavian artery (Type $B_{3,x}$^34^). Patient 1’s re-entry tear is located distal to the celiac artery and proximal to the superior mesenteric artery. The false lumen runs on the outside of the aortic arch. Patient 2’s re-entry tears are located at the level of the left internal and external iliac arteries. The false lumen runs on the inside of the aortic arch. Patient 3’s re-entry tears are located at the left renal artery and the left external iliac artery. The false lumen runs on the outside of the aortic arch and is thrombosed up to the middle of the descending thoracic aorta. A large fenestration (3.7 mm) is located distal to the thrombus.

In the FL, Patient 1 presents high blood flow velocity, an impingement area, and high TAWSS distal to the entry tear, which correlate with a dilated false lumen. Indeed, the two cross-sections of the descending thoracic aorta have a FL area 3.4x (DTA) and 2.9x (DTA2) larger than the TL area. The FL flow ratios of 57% (DTA) and 43% (DTA2) also support the dilation of the FL. As for the luminal pressure difference, it is negative $\left(\Delta P_{TL-FL}=-5.8\;mmHg\right)$ at the DTA2 cross-section. A negative luminal pressure suggests the expansion of the FL and the compression of the TL since the FL has a higher pressure than the TL^21,22^.

While the FL of Patient 2 also presents high blood flow velocity, an impingement area, and high TAWSS distal to the entry tear, it is mainly dilated at the tear and proximal to the tear. The FL area distal to the tear is 2.8x smaller than the TL area. This could be attributed to the location of the entry tear in the inner curve of the aorta, which creates a FL dilation towards the brachiocephalic trunk. Patient 2 also presents a high and positive luminal pressure difference at all cross-sections, with $△P_{TL-FL}$ = 35.6 mmHg at DTA before peak systole. This could help support the dissection flap and protect the TL from compression^21,22^. The smaller size of the entry tear (Table 1) could also contribute to the high luminal pressure difference^43^, and in turn, to a more stable FL. For Patient 3, lower FL flow velocity and TAWSS are observed in the fenestration region compared to the TL, which results in a more stable false lumen with a very low FL flow ratio of 14% at the DTA and DTA2. Two anatomical features resulting in favorable hemodynamic indices seem to correlate with more stability and less growth of the FL: (i) the inner curve orientation of the false lumen (Patient 2) and (ii) the presence of a large fenestration^20^ in the descending thoracic aorta (Patient 3). The first feature seems to maintain a positive luminal pressure difference, which protects the TL from compression. The second feature seems to reduce the magnitude of the negative luminal pressure difference, which minimizes the risk of FL expansion. The second feature is investigated using modified models of Patient 3 in the following section.

### Thrombosis and Fenestration

Anatomic features of the dissection, including the presence of fenestrations, may impact short-term and long-term TBAD outcomes. In addition, TEVAR by necessity and its nature has the effect of covering a number of fenestrations between the FL and TL. The impact of this coverage is often unpredictable in the clinical setting especially regarding its effects on perfusion for the distal organ vessels and the long-term remodeling of the TL and FL. To better investigate the effect of a large fenestration in the descending thoracic aorta and thrombosis in the FL, two additional models were created for Patient 3 neglecting the presence of the thrombus: (i) the ET model, a model without the fenestration and with an entry tear of size 11 mm × 9 mm distal to the subclavian artery, and (ii) the ET_FN model, a model with the same primary entry tear as the ET model and the same large fenestration as the original model. Hemodynamic parameters from each model were compared to investigate any significant differences. As shown in Figure 4 and 7, the general trend of the flow rate and luminal pressure difference is similar across all three models. The luminal pressure difference at DTA2 and IAA is first negative before the systolic peak, then positive at the beginning of diastole, and finally negligible. This means that the FL has a higher pressure than the TL before the systolic peak, which could lead to aneurysmal degeneration^21,22^. Adding the fenestration in the ET model to obtain the ET_FN model helps decrease FL pressurization as $\Delta P_{TL-FL}$ increases by 4.9 mmHg and it becomes less negative at DTA2. Adding the thrombus in the ET_FN model to obstruct the entry tear, as shown in the original model, also helps decrease FL pressurization as $\Delta P_{TL-FL}$ increases by 3.3 mmHg, becoming less negative. With a combination of a thrombus and a large fenestration (3.7 mm) in the descending thoracic aorta, the luminal pressure difference $\Delta P_{TL-FL}$ increases by 8.2 mmHg and becomes closer to 0. This decrease in FL pressurization supports the natural formation of a thrombus and a large fenestration in Patient 3, which might protect Patient 3 from developing an aneurysm in the false lumen. Two main differences are observed in the streamlines of Patient 3’s new models (Fig. 5 and 8). First, the impingement area in the FL distal to the entry tear has a higher flow velocity and a higher TAWSS in the ET_FN model compared to the ET model. Second, the flow accelerates in the arch to re-enter the TL through the fenestration. The latter observation could explain the former. While the presence of the fenestration reduces FL pressurization, it could lead to wall degeneration faster distal to the entry tear. This potential threat is removed by the thrombus (i.e., no flow) while also reducing FL pressurization as previously discussed.

### Limitations

In this study, inaccuracies introduced in the patient-specific flow boundary conditions were mitigated by using a patient-specific stroke volume, which can be a good proxy to impose a patient-specific inlet flow profile^11^. The baseline inlet flow rates and the outlet fractional flow rate values were obtained from Bäumler *et al.*^36^. The inlet flow rates were tuned to match the stroke volume for Patient 2 and 3. However, the lack of access to the LVOT VTI of Patient 1 prevented the computation of the corresponding stroke volume. Further inaccuracy is introduced with the uniform dissection flap and rigid-wall assumption, especially in regions of expected high deformation such as the false lumen, dissection flap, and entry tear^44^. Validation of the hemodynamic outcomes described in these results needs to be performed using corroborating studies including 4D-flow MRI scans to provide patient-specific flow and boundary conditions information. However, the models in this study were developed using CT scans typically obtained in clinical settings at onset of the patients’ acute presentation in the hospital emergency room. Therefore, validated predictions based on CT images obtained as standard of care, without the need for further studies using 4D-flow MRI information, could provide valuable and broadly accessible insights into patients’ outcomes. In particular, outcomes resulting from anatomic features of the dissection, and the hemodynamic consequences of various interventions including TEVAR, which necessarily covers a number of fenestrations in addition to the entry tear, could be explored.

## Conclusions

In this study, patient-specific CFD models have been obtained leveraging point of care CT images from three different patients suffering from acute Type B aortic dissection to investigate the role of FL thrombosis, fenestration, and false lumen orbital orientation. SimVascular^35^ was used to create anatomic models and run simulations and a custom code was developed to improve the scalability of aortic-dissection CFD analysis. In particular, this approach considerably accelerates (*i*) the tuning of the parameters to meet target pressures, (*ii*) CFD results visualization, and (*iii*) lumen cross-sectional analysis. The results show that both thrombosis in the FL at the entry tear, and the presence of a large fenestration in the thoracic aorta reduce the magnitude of the negative luminal pressure difference $\Delta P_{TL-FL}$ by 3.3 mmHg and 4.9 mmHg, respectively. Having similar luminal pressures in the TL and FL is thought to reduce the risk of FL expansion, hence promoting the formation of a stable false lumen^18,45^. Despite an impingement area in the false lumen near the entry tear, Patient 2 has a positive and high luminal pressure difference (up to $\Delta P_{TL-FL}=35.6\;mmHg$), which is thought to promote compression of the false lumen and expansion of the true lumen.^18,45^ The discrepancy between the impingement area and the high luminal pressure suggests that the orientation of the false lumen on the inner curve of the arch helps maintain a positive luminal pressure difference. This study demonstrates the potential of using CFD simulations in clinical settings to determine which anatomical features and hemodynamic characteristics are present in aortic dissections of varying severity. This approach can lead to a better understanding of the mechanisms behind TBAD progressive aortic growth dilatation and it can be leveraged to optimize treatment outcomes.

## Supplementary Material

Supplement 1

## Figures and Tables

**Table 1.** Patients’ demographics, aortic dissection morphological details, and CT image characteristics.

	Patient 1	Patient 2	Patient 3
Age	70	68	62
Sex	F	M	M
Race/Ethnicity	Hispanic/Latino	Black/African American	White
SVS/STS Description	Type *B*_3,6_	*B*_3,11_	*B*_3,11_
Entry tear size (*mm*)	12.1	5.4	Unclear
Max involved aortic diameter (cm)	4.4	3.5	3.2
Max intimal thickness (mm)	2.9	2	2.4
CT transverse section thickness (mm)	4	2	2
CT total transverse images	213	661	1635
CT pixel size (*mm*^2^)	0.98 × 0.98	0.88 × 0.88	0.74 × 0.74

## Data Availability

The data that supports the results within this paper are available from the corresponding author upon reasonable request.
