# Supplementary material for: Investigating the Role of Thrombosis, Fenestration, and False Lumen Orbital Orientation in the Hemodynamics of Type B Aortic Dissection
Source: Res Sq. 2024 Mar 15:rs.3.rs-3997160. Preprint. [Version 1] doi: 10.21203/rs.3.rs-3997160/v1 (PMC10980148; doi:10.21203/rs.3.rs-3997160/v1)
Supplement: Supplement 1 [file NIHPPRS3997160v1-supplement-1.pdf]

Supplementary Material

Table S1. Fractional flow rate at each branch outlet used to tune CFD boundary conditions[36].

Branch Name	Fraction of Flow
Brachiocephalic trunk	0.202
Left common carotid artery	0.058
Left subclavian artery	0.135
Celiac trunk	0.126
Superior mesenteric artery	0.041
Right renal artery	0.072
Left renal artery	0.084
Right external iliac	0.110
Right internal iliac	0.045
Left external iliac	0.090
Left internal iliac	0.037

Table S2. Patients' information and experiment parameters.$^{+}$Baseline inlet flow since LVOT VTI was not provided. * Relative difference with the patient's values.

	Patient 1	Patient 2	Patient 3
**Inlet Information and Parameters**			
LVOT Area (cm^2)	4.275	3.722	6.962
LVOT VTI (cm)	-	36	17
Stroke Volume (mL)	96.5^{+}	134.0	118.4
Inlet baseline scale factor	1	1.389	1.227
**Outlet Parameters**			
Total resistance R_T (g/(s·mm^4))	0.0966	0.0714	0.08736
Total capacitance C_T ($mm^4 \cdot s$/g))	12.78	14.2	15.62
Fluid density ρ_f (g/mm^3)	0.00106	0.00106	0.00106
Fluid viscocity μ_f (g/(mm·s))	0.004	0.004	0.004
Resistance Ratio	0.9	0.9	0.9
**Patient's BP and Final Model's Pressure**			
P_{sys} (mmHg)	138	149	143
P_{dia} (mmHg)	67	66	74
P_inlet_{sys} (mmHg) (rel. diff. %)*	137.4 (-0.4%)	151.3 (1.5%)	149.9 (4.8%)
P_inlet_{dia} (mmHg) (rel. diff. %)*	65.9 (-1.6%)	66.0 (0.0%)	73.5 (-0.6%)
Inlet mean pressure (mmHg) (rel. diff. %)*	89.8 (-1.0%)	94.4 (0.8%)	99 (2.1%)
Inlet pulse pressure (mmHg) (rel.. %)*	71.5 (0.7%)	85.3 (2.8%)	76.4 (10.7%)

Table S3. Cross-sections of interest flow and pressure statistics for Patient 1,2, and 3. **Significant numbers discussed in the manuscript are in bold.**

	Patient 1			Patient 2			Patient 3		
	DTA	DTA2	IAA	DTA	DTA2	IAA	DTA	DTA2	IAA
FL Area Ratio	**3.4**	**2.9**	-	**0.35**	1.19	0.92	-	**1.0**	0.63
FR_{FL} (%)	**57**	**43**	-	**34**	38	12	-	**14**	14
Min ΔP_{TL-FL} (mmHg)	-2.04	**-5.76**	-	-0.21	-0.61	-0.5	-	**-13.4**	**-18.6**
Max ΔP_{TL-FL} (mmHg)	1.86	0.71	-	**35.6**	**27.1**	**17.3**	-	**6.34**	**10.3**
Range ΔP_{TL-FL} (mmHg)	3.9	6.47	-	35.81	27.71	17.8	-	19.74	28.9

Table S4. Cross-sections of interest flow and pressure statistics for Patient 3's models (Original, ET_FN, ET). **The decreasing behavior of the luminal pressure difference ΔP_{TL-FL} at DTA2 is highlighted in bold.**

	Patient 3 - Original			Patient 3 ET_FN			Patient ET		
	DTA	DTA2	IAA	DTA	DTA2	IAA	DTA	DTA2	IAA
FL Area Ratio	-	1.0	0.63	0.77	1.0	0.63	0.77	1.0	0.63
FR_{FL} (%)	-	14	14	26	16	14	17	17	15
Min ΔP_{TL-FL} (mmHg)	-	**-13.4**	-18.6	-0.66	**-16.7**	-22.1	1.56	**-21.6**	-26.3
Max ΔP_{TL-FL} (mmHg)	-	6.34	10.3	3.01	5.35	9.08	0.55	7.39	11.0
Range ΔP_{TL-FL} (mmHg)	-	19.74	28.9	3.67	22.05	31.18	2.11	28.99	37.3

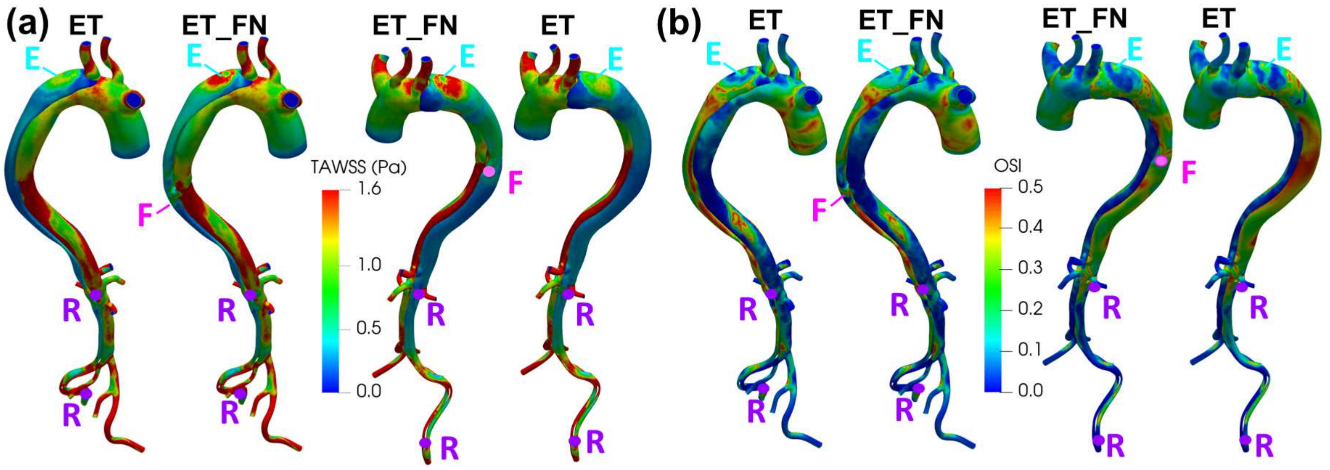

Figure S1. **(a)** TAWSS and **(b)** OSI distributions of the ET and ET_FN models of Patient 3. (E) and (R) mark the entry and re-entry tear(s) locations, (F) marks the location of a large fenestration in the descending thoracic aorta. The false lumen is the channel along the outer curvature of the aorta in all views.
